# Dysregulation of Ki-67 Expression in T Cells of Children with Autism Spectrum Disorder

**DOI:** 10.3390/children8020116

**Published:** 2021-02-06

**Authors:** Khaled Alhosaini, Mushtaq A. Ansari, Ahmed Nadeem, Sabry M. Attia, Saleh A. Bakheet, Laila Y. Al-Ayadhi, Hafiz M. Mahmood, Haneen A. Al-Mazroua, Sheikh F. Ahmad

**Affiliations:** 1 Department of Pharmacology and Toxicology, College of Pharmacy, King Saud University, Riyadh 11451, Saudi Arabia; kalhosaini@ksu.edu.sa (K.A.); muansari@ksu.edu.sa (M.A.A.); anadeem@ksu.edu.sa (A.N.); attiasm@ksu.edu.sa (S.M.A.); sbakheet@ksu.edu.sa (S.A.B.); harshad@ksu.edu.sa (H.M.M.); halmazroua@ksu.edu.sa (H.A.A.-M.); 2 Autism Research and Treatment Center, AL-Amodi Autism Research Chair, Department of Physiology, College of Medicine, King Saud University, Riyadh 11451, Saudi Arabia; lyayadhi@ksu.edu.sa

**Keywords:** autism spectrum disorder, typically developing control, Ki-67, peripheral blood mononuclear cells, inflammatory mediators

## Abstract

Autism spectrum disorder (ASD) is a neurodevelopmental disorder characterized by behavioral abnormalities such as impairments in social function and deficits in communication. The etiology of autism is unknown in most cases, but many studies have pointed towards the immune system as a causative agent in autism. Specific studies implicated lymphocytes, natural killer (NK) cells, monocytes, cytokines, and specific transcription factors in the development of ASD. The protein Ki-67 is n expressed in the proliferating cells and is used as a tool in several disorders. Ki-67 plays a crucial role in many neurological diseases. However, Ki-67 role in ASD is not fully understood. In this study, we investigated the possible role of Ki-67 expression in autistic children. We compared Ki-67 production in CD3+, CD4+, CD8+, CXCR4+, CXCR7+, CD45R+, HLA-DR+, GATA3+, Helios+, and FOXP3+ peripheral blood mononuclear cells (PBMCs) in autistic children to typically developing (TD) controls using immunofluorescence staining. We also determined Ki-67 mRNA levels in PBMCs using RT–PCR. The results revealed that autistic children had significantly increased numbers of CD3+Ki-67+, CD4+Ki-67+, CD8+Ki-67+, CXCR4+Ki-67+, CXCR7+Ki-67+, CD45R+Ki-67+, HLA-DR+Ki-67+, CXCR4+GATA3+, GATA3+Ki-67+ cells and decreased Helios+Ki-67+ and FOXP3+Ki-67+ cells compared with TD controls. In addition, the autistic children showed upregulation of Ki-67 mRNA levels compared with TD controls. Further studies need to be carried out to assess the exact role of Ki-67 and its therapeutic potential in ASD.

## 1. Introduction

Autism spectrum disorder (ASD) is a group of complex multifactorial neurodevelopmental and behaviorally defined conditions [1] characterized by deficits in social communications and the presence of restricted, repetitive behavioral patterns, interests, or activities [2]. Abnormal immune responses have been demonstrated in children with ASD [3]; autistic children have several immune phenotypes that correlate with increasingly severe behavioral impairments [3,4]. However, the complicated relationship between the immune system and autism symptomatology is unclear [5,6].

Alterations in the peripheral immune system of individuals with ASD cause immune system dysregulation [7]. Individuals diagnosed with ASD have altered cytokine profiles [8], and chemokine receptors play an important role in neurological disorders [9]. We previously reported that immune dysfunction is caused by an imbalance in inflammatory mediators and transcription factor signaling in autistic children [10,11]. Furthermore, increased chemokine receptor activation plays an essential role in immune dysfunction in autistic children, and chemokine receptor expression is upregulated in the peripheral and brain tissue of autistic individuals [12,13]. Increased levels of chemokine receptors have also been linked with behavioral deficits in autistic children [14]. Activation of the Janus kinase (JAK)-signal transducer and activator of transcription (STAT) signaling pathway may also play an essential role in immune dysfunction in autistic individuals [15].

Altered activation of specific immune cells, including T lymphocyte cell production, occurs in autistic individuals [16,17,18]. Previous studies indicate that changes in Human Leukocyte Antigen – DR isotype (HLA-DR), a marker of T-cell activation, are associated with ASD [19,20]. In addition, immune-activated mothers’ offspring showed a systemic impairment in regulatory T (Treg) cells [21]. However, the exact mechanisms underlying these developments requires further investigation.

Ki-67 is expressed in proliferating cells, so it can be used as a marker for proliferation. Ki-67 is also used as a tool in different cancer types [22,23]. Importantly, in breast tumors, high Ki-67 levels are associated with a higher risk of central nervous system (CNS) metastases and cancer progression [23,24]. Ki-67 was significantly higher in individuals with ASD compared with age-matched controls [25]. The GATA3 transcription factor regulates sympathetic neuron development [26]. Increasing levels of GATA3 in PC-12 cells triggered ASD development [27]. In this study, we hypothesized that Ki-67 expression could be dysregulated in immune cells of children with ASD.

## 2. Material Methods

### 2.1. Ethics Approval

The local ethics committee of the Faculty of Medicine, King Saud University, Riyadh, Saudi Arabia, approved this human study (Approval# E-10-220). Informed written consent for participation in the study was signed by the parents or the legal guardians of the subjects.

### 2.2. Study Participants

A cross-sectional study was conducted on 40 male children with classic-onset ASD who were enrolled in the study over a period of 9 months. Subjects fulfilled the standards for the diagnosis of autism according to the 5th edition of the Diagnostic and Statistical Manual of Mental Disorders [2]. Their ages ranged from 4 to 12 years (mean ± SD = 5.1 ± 2.47 years). All ASD subjects were examined for tuberous sclerosis, Angelman syndrome, dysmorphic features, or any other neurological disease. Data on current and past physical illnesses were collected through parental interviews.

The typically developing controls (TD) group was comprised of 30 age- and sex-matched healthy male children. The healthy siblings of the healthy infants who appeared at the Well Baby Clinic, King Khalid University Hospital for routine follow-up were enrolled. The children were not related to the ASD patients, and no clinical findings suggestive of neuropsychiatric disorders were observed. Their ages ranged from 4 to 12 years (mean ± SD = 5.3 ± 2.17 years). Well-versed written agreements to participate in this study were given by the parents or legal guardians of the study subjects.

### 2.3. Study Measurements

Clinical evaluations of ASD subjects were established based on clinical history, clinical examination, and neuropsychiatric assessment. The severity of disease was measured using the childhood autism rating scale (CARS), which rates the child on a scale from one to four in each of sixteen areas (relating to people; verbal and non-verbal communications, emotional response, imitation, body use, listening response, fear or nervousness, adaptation to change, taste, activity level, touch and smell response, visual response, level and consistency of intellectual response, and general impressions) [28].

### 2.4. Chemicals and Antibodies

Fluoroisothiocyanate, PE/dazzle, allophycocyanin, and phycoerythrin-labeled CD3, CD4, CD8, CXCR4, CXCR7, CD45R, HLA-DR, GATA3, Ki-67, Helios, and FOXP3 human monoclonal antibodies, RBC lysis buffer (10X), intracellular staining permeabilization wash buffer (10X), and fixation buffer were purchased from BioLegend (San Diego, USA). GolgiStop was purchased from BD Biosciences (San Diego, USA). RPMI 1640 medium, phorbol myristate acetate (PMA), and ionomycin were purchased from Sigma-Aldrich (St. Louis, MO, USA). TRIzol was purchased from Life Technologies (Paisley, UK). SYBR green and cDNA kits were purchased from Applied Biosystems (Foster City, CA, USA). Primers were synthesized from GenScript (Piscataway, NJ, USA).

### 2.5. Flow Cytometric Analysis

Human peripheral blood mononuclear cells (PBMCs) were isolated using density-gradient centrifugation as previously described (Ahmad et al., 2017). Flow cytometric analysis was performed to assess Ki-67 production in CD3+, CD4+, CD8+, CXCR4+, CXCR7+, CD45R+, HLA-DR+, GATA3+, Helios+, and FOXP3+ cells. Briefly, PBMCs were stimulated for 4 h with PMA/ionomycin (Sigma-Aldrich, St. Louis, MO, USA) in the presence of GolgiStop ((BD Biosciences, San Jose, CA, USA)) as previously described [10,29]. PBMCs were washed, and anti-CD3, anti-CD4, anti-CD8, anti-CXCR4, anti-CXCR7, anti-CD45R, and anti-HLA-DR (BioLegend, San Diego, CA, USA) staining was performed. Cells were fixed and permeabilized for staining with anti-Ki-67, anti-GATA3, anti-Helios, and anti-FOXP3 (BioLegend) antibodies. Forward scatter/side scatter and single-cell gating were used to exclude dead cells from all analyses. Data were acquired and analyzed with an FC 500 flow cytometer Beckman Coulter (Indianapolis, IN, USA) using CXP software (Beckman Coulter, USA).

### 2.6. Gene Expression

RNA was extracted from PBMCs using Trizol reagent and quantified according to a previously described method [10,29]. RNA concentration was determined with a Nanodrop spectrophotometer (Thermo Scientific, Wiggins Ave, Bedford, MA, USA). The cDNAs were synthesized using a high-capacity cDNA reverse transcription kit. Quantitative RT–PCR was performed using SYBR Green master mix, as previously described [10,11,29]. The primers used in the assay were as follows: Ki-67, F: 5′-GGATCGTCCCAGTGGAAGAG-3′, R: 5′-TCTCGTGGGCCACATTTTCT-3′; GAPDH, F: 5′-AATGGGCAGCCGTTAGGAAA-3′, R: 5′-GCGCCCAATACGACCAAATC-3′. Data are presented as the fold of change in mRNA expression normalized to GAPDH.

### 2.7. Statistics

The investigated parameters were first tested for normality using Shapiro–Wilk’s test. All studied parameters showed normal distribution of data. Student’s *t*-tests were used for statistical comparison between two groups. All statistical tests are indicated in figure legends. Statistical analyses were performed using GraphPad Prism statistical package. The data were expressed as mean ± SD. *p* values <0.05 were considered significant.

## 3. Results

### 3.1. Increased Ki-67 Expression in T Cell Surface Receptor+ Cells in Children with ASD

**Figure 1:**
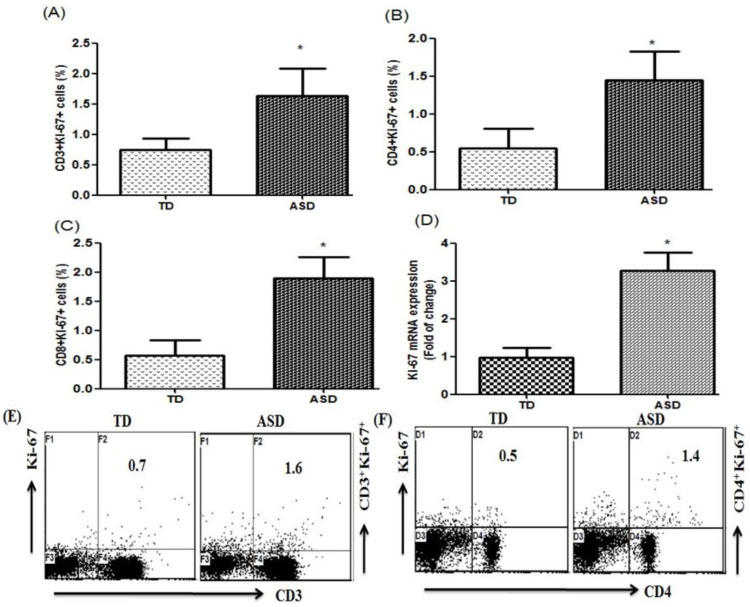
Ki-67 levels in CD3+, CD4+, and CD8+ T peripheral blood mononuclear cells (PBMCs). (**A**–**C**) flow cytometric analysis of Ki-67-producing CD3+, CD4+, and CD8+ T cells, respectively, from PBMCs in autism spectrum disorder (ASD) and typically developing (TD) controls. (**D**) The mRNA expression level of Ki-67 in PBMCs measured using quantitative RT–PCR and normalized to); glyceraldehyde-3-phosphate dehydrogenase (GAPDH). (**E**,**F**) Representative flow cytometry dot plots showing the percentage of Ki-67-producing CD3+ and CD4+ T cells from TD controls and children with ASD. Statistical analysis was carried out by Student’s *t*-test. Statistical significance was determined as * *p* < 0.05.

The number of Ki-67-producing CD3+ cells in children with ASD and TD controls in PBMCs was evaluated with flow cytometry. As shown in Figure 1A, Ki-67-producing CD3+ cells elevated in children with ASD compared with TD controls (*t* = 4.538, *p* < 0.001). Ki-67-producing CD4+ and CD8+cells also increased in children with ASD as compared with TD controls (Figure 1B; *t* = 4.186, *p* < 0.001 and C; *t* = 7.381, *p* < 0.001). Additionally, Ki-67 mRNA expression increased in the PBMCs from children with ASD as compared with the TD controls (Figure 1D; *t* = 10.53, *p* < 0.001) control group. These results suggest that Ki-67 plays an important role in ASD.

### 3.2. Increased Ki-67 Expression in CXCR4 and CXCR7+ Cells in Children with ASD

**Figure 2:**
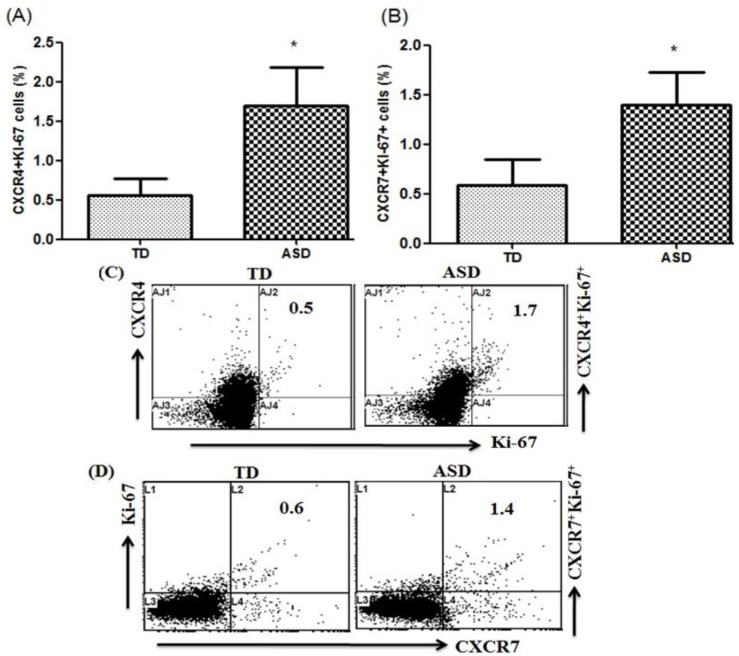
Ki-67 expression in CXCR4+ and CXCR7+ peripheral blood mononuclear cells (PBMCs). (**A**,**B**) Flow cytometric analysis of Ki-67-producing CXCR4+ and CXCR7+ (chemokine receptors) PBMCs in children with autism spectrum disorder (ASD) and typically developing (TD) controls. (**C**,**D**) Representative flow cytometry dot plots showing the percentage of Ki-67-producing CXCR4+ and CXCR7+ PBMCs from TD controls and children with ASD. Statistical analysis was carried out by Student’s *t*-test. Statistical significance was determined as * *p* < 0.05.

To elucidate the role of chemokine receptors, we measured the percentage of Ki-67-expressing CXCR4+ and CXCR7+ cells in children with ASD compared to TD controls (Figure 2A; *t* = 5.25, *p* < 0.001). The percentage of Ki-67-expressing CXCR4+ and CXCR7+ cells significantly increased in children with ASD compared with TD controls (Figure 2B; *t* = 4.742, *p* < 0.001). Our results suggest that both CXCR4 and CXCR7 play an important role in ASD and are potential clinically related disease markers of ASD.

### 3.3. Ki-67 Production Is Upregulated in CD45R+ and HLA-DR+ Cells in Children with ASD

**Figure 3:**
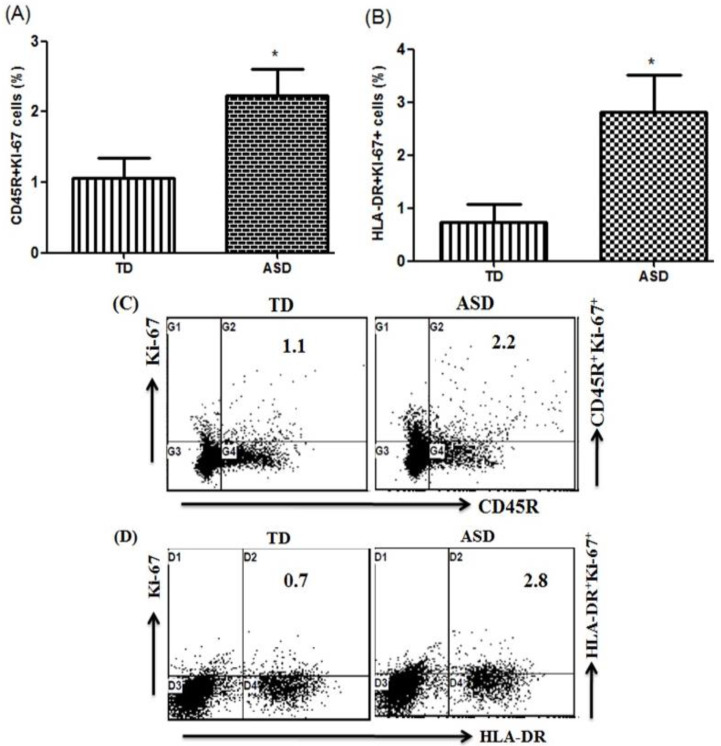
Ki-67 expression in CD45R+ and HLA-DR+ peripheral blood mononuclear cells (PBMCs). (**A**,**B**) Flow cytometric analysis of Ki-67-producing CD45R+ and HLA-DR+ PBMCs in children with autism spectrum disorder (ASD) and typically developing (TD) controls. (**C**,**D**) Representative flow cytometry dot plots showing the percentage of Ki-67-producing CD45R+ and HLA-DR+ PBMCs from TD controls and children with ASD. Statistical analysis was carried out by Student’s *t*-test. Statistical significance was determined as * *p* < 0.05.

Ki-67 expression in CD45R+ and HLA-DR+ cells of children with ASD and TD controls in PBMCs was evaluated with flow cytometry. As shown in Figure 3A, the number of CD45R+Ki-67+ cells increased significantly in children with ASD compared with the TD controls (*t* = 6.07, *p* < 0.001). Furthermore, Ki-67-producing HLA-DR+ cells also increased significantly in children with ASD compared with the TD controls (Figure 3B; *t* = 6.564, *p* < 0.001). These results suggest that Ki-67 expression is upregulated in children with ASD.

### 3.4. GATA3, Helios, and FOXP3 Transcription Factor Expression in Ki-67 Producing Cells in Children with ASD

**Figure 4:**
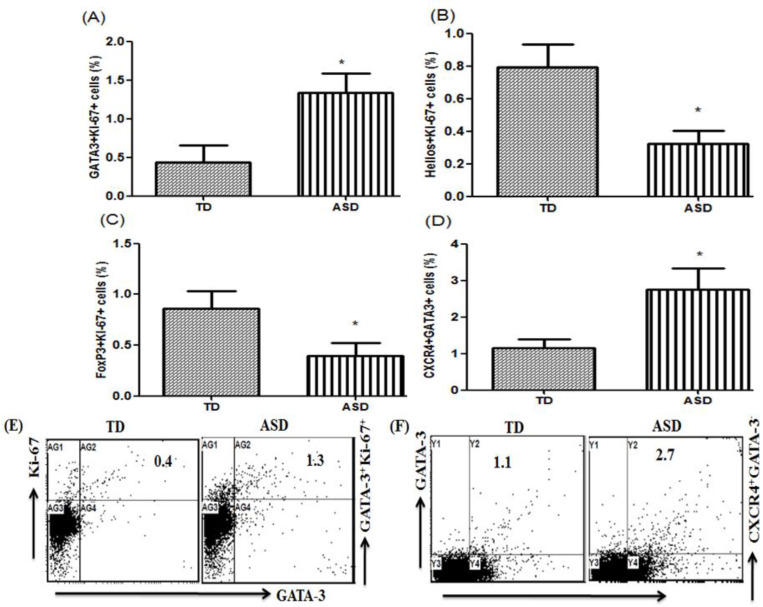
Ki-67 expression in GATA3+, Helios+, and FOXP3+ peripheral blood mononuclear cells (PBMCs). (**A**–**C**) Flow cytometric analysis of intracellular Ki-67-producing GATA3+, Helios+, and FOXP3+ PBMCs in children with autism spectrum disorder (ASD) and typically-developing (TD) controls. (**D**) Flow cytometric analysis of GATA-3 production in CXCR4+ cells. (**E**,**F**) Representative flow cytometry dot plots showing the percentage of Ki-67-producing GATA3+ and CXCR4+GATA3+ PBMCs from TD controls and children with ASD. Statistical analysis was carried out by Student’s *t*-test. Statistical significance was determined as * *p* < 0.05.

The association of transcription factors with Ki-67+ cells was analyzed using flow cytometry in PBMCs of children with ASD and TD controls. Ki-67-producing GATA3+ cells were significantly higher in children with ASD than TD controls (Figure 4A; *t* = 6.508, *p* < 0.001). In contrast, children with ASD had significantly fewer Ki-67-producing Helios+ cells and Ki-67-producing FOXP3+ cells compared with TD controls (Figure 4B; *t* = 7.162, *p* < 0.001 and C; *t* = 5.321, *p* < 0.001). The number of GATA3 cells expressing CXCR4+ was evaluated; the number of CXCR4+GATA-4+ cells increased in children with ASD compared with TD controls (Figure 4D; *t* = 6.244, *p* < 0.001). These results provide evidence that these transcription factors could also be a key indicator of immune alterations in ASD.

## 4. Discussion

Autistic physiopathology involves several modifications at genetic and immune levels, including increased proinflammatory mediators [30]. Different factors, including transplacental antibodies, maternal immune activation, and congenital infections, play a crucial role in the pathophysiology of ASD [6]. Children with ASD have also shown the linkage between immune dysregulation and behavioral impairments [31,32]. Furthermore, ASD is associated with abnormal immune function, including cytokine dysregulations, inflammation, and autoantibodies presence. It has been reported in previous studies that anti-brain immunoglobulins exist in individuals with ASD [33,34]. There is a direct correlation of systemic cytokines with the severity of disease in autism, which may contribute to the proinflammatory environment [35,36].

Ki-67 may be involved in immune dysfunction. Sp1-binding sites are necessary for the regulation of Ki-67 transcription [37]. In a previous report, Ki-67+ cells were higher in ASD individuals compared to age-matched controls [25]. Further, elevated Ki-67+ cells were seen in the rheumatoid arthritis pannus invading hard tissues [38]. This study assessed Ki-67 expression in peripheral immune cells such as T cells, which were previously unexplored in ASD subjects. Our present analysis of Ki-67-producing T cell receptor-positive PBMCs revealed remarkably statistically significant increases in Ki-67+ cells in autistic children compared to the TD controls. The present study also demonstrated that Ki-67 mRNA expression increased in ASD children compared with TD controls. These results suggest that the upregulation of Ki-67 expression is associated with disease manifestation during ASD. This observation indicates that activation of Ki-67 cells in immune cells in ASD individuals may be associated with behavioral features of ASD. However, more studies are required to determine the role of Ki-67 in ASD subjects.

Previous studies evaluated the function of chemokine receptors in neurodevelopmental/neuropsychiatric disorders. Earlier research reported a significant contribution of chemokine receptors in promoting the T-cell activation in neuronal cells [39] and demonstrated the functional expression of CXCR4 on epithelial cells in humans [40]. CXCR7 is expressed in neurons, astrocytes, microglia, and endothelial cells of the brain [41]. The pattern of CXCR7 expression suggests that it plays a central role in CNS development [42]. Our results displayed a significant increase in Ki-67-producing CXCR4+ cells in ASD children compared with TD controls. Similarly, ASD subjects also showed noticeable induction of Ki-67-producing CXCR7+ cells. Our results indicate that the induction of CXCR4+Ki-67+ and CXCR7+Ki-67+ cells may be involved in immune dysregulation that is observed in ASD.

HLA-DR expression is a susceptibility marker for several autoimmune disorders, including autoimmune diabetes, hypothyroidism, and rheumatoid arthritis; these disorders are risk factors in ASD [43,44]. Furthermore, increased HLA-DR expression in pregnant women is a risk factor for ASD in newborn children [45]. A study showed that HLA-DR4 alleles occurred in ASD individuals [19]. The transmission disequilibrium test suggested that HLA-DR is linked to ASD [20]. The number of Ki-67-expressing HLA-DR+ cells was elevated in ASD subjects compared with TD controls. Moreover, we showed that Ki-67-expressing CD45R+ cells were elevated in autistic children. These results show that the elevation of Ki-67+HLA-DR+ and Ki-67+CD45R+ cells in children with ASD may play a role in the modulation of behaviors and core features of ASD.

Elevated GATA3 expression is involved in developing serotonergic neurons in the caudal raphe nuclei [46]. Increased GATA3 levels induced by three teratogens in PC-12 cells causes ASD [27]. In a recent study, GATA3 regulated the transcriptional activity of dopamine β-hydroxylase [47]. Expression of GATA3 was detected in the raphe nuclei, mid-brain, and pretectal regions of the CNS [48]. Our results revealed that GATA3+Ki-67+ production increased in children with ASD, suggesting that behavior and communication deficit in children with ASD are related to the upregulation of GATA3+Ki-67+ expression. Further studies are to be required to elaborate on the role of GATA3 signaling in ASD individuals.

Helios is a marker for Treg cell differentiation [49]. Helios defines T cell tolerance in the periphery and thymus [50]. The percentage of FOXP3+Helios+ Treg cells positively correlates with the activity of the disease in systemic lupus erythematosus [51]. Our data showed that Helios+Ki-67+ cells were significantly diminished in ASD subjects, which may be linked with immune dysfunction in these individuals. Recently, we showed that children with autism have fewer FOXP3+ cells [11]. FOXP3 plays an essential role in counterbalancing the overactive immune system, and its deficiency is linked to autoimmune diseases and ASD [21]. Decreased FOXP3 gene variations increase susceptibility to ASD [52]. In this study, we display that Ki-67-producing FOXP3+ cells have diminished % in ASD subjects as compared to TD children. The reduction of Ki-67+FOXP3+ cells may cause a higher vulnerability for immune dysregulations in ASD subjects with autism. Further studies are required to understand the role of Ki-67+FOXP3+ cells in ASD subjects.

## 5. Conclusions

Our results demonstrate increased Ki-67 production/expression in children with ASD, indicating that Ki-67 may be associated with immune dysregulation. Our results provide important information about the immune cells having dysregulated Ki-67 expression along with other immune markers in ASD. Further studies need to be conducted before it becomes evident whether Ki-67 is a possible molecular target relevant to the therapeutic and etiological aspects of ASD.

## Data Availability

The authors confirm that all data underlying the findings are fully available without restriction. All relevant data are within the paper.

## Abbreviations

Autism spectrum disorder (ASD); typically developing (TD); peripheral blood mononuclear cells (PBMCs); messenger RNA (mRNA); reverse transcription polymerase chain reaction (RT–PCR); C-X-C chemokine receptor type 4 (CXCR-4); C-X-C chemokine receptor type 7 (CXCR-7); human leukocyte antigen—DR isotype (HLA-DR); forkhead box P3 (Foxp3); GATA3 (GATA-binding protein 3); regulatory T (Treg); childhood autism rating scale (CARS); phorbol myristate acetate (PMA); phycoerythrin (PE); allophycocyanin (APC); fluoroisothiocyanate (FITC); glyceraldehyde-3-phosphate dehydrogenase (GAPDH); complementary DNA (cDNA).
